# Transition cow nutrition for grazing herds: Practical priorities for health and performance

**DOI:** 10.3168/jdsc.2025-0984

**Published:** 2026-05-01

**Authors:** Mallory Crookenden, Axel Heiser

**Affiliations:** AgResearch Group, Bioeconomy Science Institute, Christchurch, New Zealand 7608

## Abstract

The transition period, encompassing the 3 wk before and 3 wk after calving, represents the most metabolically and immunologically demanding phase of the dairy cow's lactation cycle. Despite lower milk yield targets, grazing cows experience homeorhetic adaptations and immune perturbations comparable in magnitude to those of high-yielding, TMR-fed cows. However, pasture-based systems impose distinct management constraints that necessitate a pragmatic, rather than precision-focused, approach to nutritional management. This review synthesizes recent evidence on transition physiology in grazing systems and examines strategies that account for inherent pasture variability while maximizing on-farm achievability. The evidence demonstrates that optimal BCS at calving, controlled prepartum energy restriction to 80% to 100% of requirements, and focused mineral supplementation, particularly magnesium and calcium, remain the most cost-effective determinants of transition success. Secondary strategies, including targeted fatty acid supplementation, may provide additional benefits but should complement, not replace, foundational management. Emerging precision technologies offer promise for identifying individual cows at risk before clinical disease manifests, yet their utility in grazing systems depends on integration with variable pasture quality and realistic labor constraints. This review emphasizes that pragmatic, fundamentals-first management, aligned with seasonal pasture dynamics and farm-specific constraints, offers the most reliable pathway to improved transition outcomes, reduced metabolic disease, and sustainable dairy production in grazing systems.

## Highlights

• Optimal BCS at calving is the best predictor of positive transition outcomes.
• Moderate controlled energy (80-100%) prepartum improves health.
• Magnesium and calcium supplementation is critical in the transition period.
• Overfeeding prepartum increases risk of metabolic disorders.
• Focus on fundamentals over complex precision strategies.

## Graphical Abstract

This review explores transition cow nutrition in pasture-based dairy systems and focuses on practical strategies to improve health and productivity. The transition period (3 weeks before to 3 weeks after calving) is characterized by major metabolic and immune challenges driven by increased nutrient and metabolic demands, resulting in negative energy balance. Evidence demonstrates that grazing cows experience similar physiological stress to housed cows, but management must be adaptable to pasture variability. The most consistent determinants of success are achieving optimal body condition score (BCS) at calving, controlling prepartum energy intake (about 80-100% of requirements), and providing targeted mineral supplementation, particularly magnesium and calcium. Moderate prepartum energy restriction improves metabolic health and immune function, whereas excessive energy or insufficient BCS increases disease risk. The review emphasizes prioritizing these core management practices over complex interventions, with emerging technologies and supplements offering secondary benefits when practical and economically justified. Created in BioRender.com. Ross, M. (2026) https://biorender.com/4c8h6op

**Figure:**
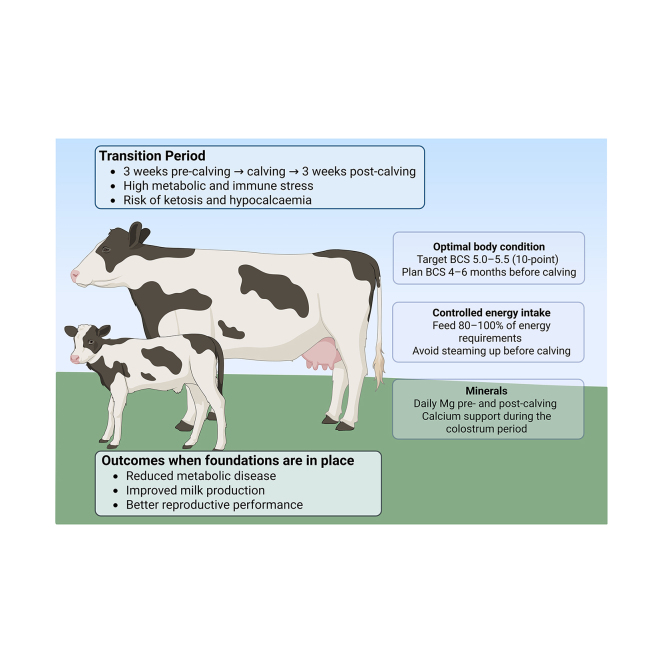

The transition from late gestation to early lactation in dairy cows represents a major metabolic and immunological challenge. Within 24 h of calving, nutrient requirements for lactation rise to roughly 2.5 to 3 times pregnancy requirements (Bell, 1995), whereas DMI increases by only 30% to 50% (Bertics et al., 1992), creating an obligate period of negative energy balance that, if managed poorly, predisposes cows to metabolic and infectious disease and contributes substantially to premature culling and economic loss (Curtis et al., 1983; Goff, 2006; Miller et al., 2008).

Historically, veterinary and nutritional advice has emphasized maximizing prepartum energy intake (“steaming up”), on the assumption that high prepartum intakes would ameliorate postpartum metabolic stress. However, over the past 15 to 20 years, controlled experimental work has contradicted this paradigm, with multiple trials in both TMR-fed and pasture-based systems showing that moderate prepartum energy restriction, compared with ad libitum feeding, improves postpartum metabolic health, energy balance, and often milk production (Roche et al., 2013a). This evidence has prompted a shift from pursuing maximally high prepartum intakes toward managing appropriate prepartum energy supply and body condition that better reflect the cow's inherent adaptive capacity.

Grazing systems present unique challenges and opportunities for transition management. On pasture-based farms, regardless of herd size, cows typically receive less individual attention per cow and feed cannot be precisely formulated on a daily basis, which is in contrast to housed systems, where TMR enable precise nutrient delivery. The key to successful transition in grazing systems lies less in matching the precision achievable in housed systems and more in correctly identifying the few critical control points that reliably determine success and aligning whole-farm feed planning. Recent molecular evidence from both grazing and housed systems has elucidated physiological mechanisms linking management decisions to health and production outcomes, with temporal gene expression profiles in liver and neutrophils demonstrating that the degree and timing of negative energy balance, BCS at calving, and prepartum feeding level directly modulate metabolic and immune adaptation capacity during the transition period (Loor et al., 2006; Crookenden et al., 2016). This deeper understanding has reinforced the primacy of a few robust management levers: achieving optimal BCS at calving, supplying adequate but not excessive prepartum energy, and providing focused mineral supplementation; refinements beyond these fundamentals should be judged by their on-farm feasibility and economic return in grazing systems (Roche, 2023).

The purpose of this narrative mini-review is to synthesize current evidence on transition nutrition in pasture-based dairy systems, with emphasis on distinguishing what is well supported from what remains uncertain and on identifying practices that are realistically implementable on commercial grazing farms. The review was undertaken by a structured search of PubMed (https://pubmed.ncbi.nlm.nih.gov/), CAB Abstracts (via Web of Science platform), and Web of Science (Clarivate, https://www.webofscience.com/) for English-language articles for transition cow nutrition published from 2000 to January 2026, with prioritization of studies using pasture-based grazing systems. Housed-system studies were included only where grazing evidence was limited, and their inclusion is stipulated in the text. Grazing systems were defined as those that rely on forage produced on-site and must not include grains, with most of the work cited in this review undertaken in New Zealand, where dairy production is dominated by temperate, pasture-based, predominantly seasonal-calving herds and grazed perennial ryegrass–clover supplies most of the annual metabolizable energy (Moscovici Joubran et al., 2021). The principles are applicable across grazing systems around the world; for example, homegrown perennial ryegrass supplies the majority of the diet of lactating cows in southern Australia, with shoulder seasons shifting the forage composition (Chapman et al., 2008). Further, perennial pastures are the base of pasture-based dairy in Uruguay; the level of system intensity (low compared with high input) demonstrates similar environmental impacts when well-managed (Loza et al., 2026). The primary recommendations for a well-managed pasture-based system, as discussed in this review, focus on target BCS at calving and controlled prepartum energy intakes close to requirements, with considered supplementation of magnesium and calcium.

The transition from pregnancy to lactation involves coordinated, synchronized shifts in metabolism and immune function across multiple tissues, a process termed homeorhesis to distinguish it from moment-to-moment homeostatic regulation (Bauman and Currie, 1980). These adaptations are initiated weeks before calving and persist through early lactation, with hormonal and gene expression changes that reprioritize nutrients toward the mammary gland.

Hepatic adaptations during transition are among the most pronounced. Gluconeogenic enzyme expression, including phosphoenolpyruvate carboxykinase and glucose-6-phosphatase, increases before and after calving to support the mammary gland's large glucose demand for lactose synthesis (Ingvartsen, 2006; Loor et al., 2006; White et al., 2016). Simultaneously, fatty acid oxidation pathways and ketogenic enzymes are upregulated to process the surge in nonesterified fatty acids (**NEFA**) mobilized from adipose tissue (Roche et al., 2013b). These changes are reflected in circulating metabolites: in moderate-yielding grazing cows, blood NEFA typically rise from ~0.30 mmol/L at 30 d prepartum to ~0.86 mmol/L 1 wk postpartum, with BHB increasing from ~0.65 to 0.96 mmol/L over the same interval (Spaans et al., 2022).

Adipose tissue undergoes profound functional remodeling, characterized by suppression of lipogenic gene expression and enhanced lipolytic capacity (Sumner-Thomson et al., 2011; Ji et al., 2012). This shift is mediated by decreased circulating insulin and enhanced sensitivity to adrenergic stimuli, allowing sustained mobilization of fat stores to support the energy and gluconeogenic needs of early lactation (McNamara, 1991). The efficiency of this adaptation is largely determined by prepartum BCS and the degree of negative energy balance (Roche et al., 2009; Kay et al., 2015), with cows calving at excessively high BCS mobilizing more fat, accumulating more hepatic lipid, and facing higher risk of ketosis and impaired liver function (Rasmussen et al., 1999; Akbar et al., 2015).

Immune function alterations occur in parallel. Rather than uniform “suppression,” recent evidence indicates a coordinated shift from a predominantly proinflammatory state prepartum to a more anti-inflammatory profile immediately postpartum, which is mediated by changes in cortisol, interleukins, and acute-phase proteins (Wankhade et al., 2017; Contreras et al., 2018). Gene expression analyses of circulating neutrophils from grazing cows show marked temporal changes in immune-related genes during the first weeks of lactation (Crookenden et al., 2016, 2019), and cows fed controlled prepartum energy levels (~65%–90% of requirements) exhibit enhanced expression of genes associated with immune competence compared with overfed cows at 120% of requirements (Lange et al., 2019); in grazing cows specifically, neutrophil gene expression profiles differ between cows offered 75% versus 125% of maintenance energy, with controlled feeding associated with more robust inflammatory and oxidative stress response pathways (Crookenden et al., 2017).

Collectively, this evidence indicates that metabolic and immune adaptations around calving are active, coordinated processes that can be modulated by prepartum feeding and body condition. These adaptations occur similarly in both high-yielding and moderate-yielding cows (Kay et al., 2015), implying that the physiological challenges of transition are conserved across production systems and underscoring the importance of appropriate transition management in all dairy herds.

For much of the twentieth century, the dominant extension message for transition nutrition management was to “prime” or “steam up” the cow in the final weeks of gestation, with recommendations to feed high levels of concentrates above maintenance energy requirements. This message was supported in pasture-based systems due to research undertaken in New Zealand in the 1970s by controlling pasture availability, which resulted in 20% to 25% greater milk fat in cows offered 25% over maintenance requirements (Hutton and Parker, 1973). However, controlled experiments beginning in the early 2000s systematically challenged this logic in order to minimize postpartum NEFA elevation and hyperketonemia. In New Zealand, a positive linear relationship between prepartum DMI and early postpartum liveweight loss has been demonstrated, and cows restricted to 75% to 80% of energy requirements prepartum have lower hepatic triacylglycerol, lower postpartum BHB, and reduced incidence of clinical ketosis and milk fever compared with ad libitum-fed cows (Roche et al., 2005, 2015). Similar findings were reported in North American (Dann et al., 2006; Douglas et al., 2006) and European (Agenäs et al., 2003; Holtenius et al., 2003) studies, where moderate prepartum energy restriction did not compromise postpartum performance and often improved metabolic health indices.

Mechanistic work has clarified these responses. Cows restricted to roughly 75% to 90% of energy requirements prepartum enter early lactation with enhanced hepatic gluconeogenic and fatty acid oxidation capacity, effectively, “training” the liver for the metabolic demands of lactation (Ingvartsen, 2006; Loor et al., 2006). Work in pasture-based systems repositioned the paradigm, recommending controlled prepartum energy intake at ~80% to 100% of calculated requirements and suggesting that cows should begin lactation in a controlled, rather than exaggerated, negative energy balance (Roche et al., 2010; Kay et al., 2015).

This recommendation is most advantageous for cows at appropriate prepartum BCS and requires nuance at the extremes. Cows calving at low BCS (e.g., <2.5 on a 5-point scale or <4.5 on a 10-point scale; Roche et al., 2004) may not tolerate further restriction and risk prolonged postpartum negative energy balance, with reduced milk yield and delayed reproductive recovery (Roche et al., 2009), whereas very high-BCS cows (>3.5) mobilize large NEFA stores regardless of prepartum intake and can respond poorly to severe restriction if hepatic oxidative capacity is limited by triacylglycerol accumulation (fatty liver), potentially exacerbating ketosis risk (Roche et al., 2009; Akbar et al., 2015). In practice, preferential management of BCS (3–3.25 on a 5-point scale, or 5–5.5 on a 10-point scale) remains the foundation, and controlled prepartum energy feeding is a secondary tool applied once BCS at calving is within target range.

In grazing systems specifically, controlled prepartum feeding can be achieved through pasture area allocation, incorporation of lower-energy forages, such as silages, hay, or straw, or temporary housing with a controlled TMR during the final 2 to 3 wk before calving, depending on farm infrastructure and labor (Roche, 2023). Excessive restriction of feed at less than ~70% of energy requirements should be avoided because it reduces milk yield and prolongs postpartum metabolic stress (Roche et al., 2017), whereas a moderate middle ground of roughly 80% to 100% of energy requirements for the final 3 to 4 wk before calving, adjusted for prepartum BCS, appears to balance metabolic health and production (Roche et al., 2013b).

Of all management factors influencing pasture-based transition cow outcomes, BCS at calving has emerged as the most robust and consistent predictor of postpartum health, milk production, and reproductive performance (Roche et al., 2009; Kay et al., 2015). Appropriate BCS at calving moderates the severity of postpartum negative energy balance, influences inflammatory and immune responses, and improves the likelihood of timely recovery of body reserves and fertility.

Syntheses of grazing-system data indicate that mature cows should calve at a BCS of ~3.0 (on a 5-point scale, or 5.0 on a 10-point scale), and first-lactation animals at ~3.25 (5-point, or 5.5 on a 10-point scale), because cows in this range have near maximum milk production and demonstrate the lowest incidence of metabolic disorders and better reproductive outcomes compared with cows outside these BCS (Roche et al., 2009; Akbar et al., 2015; Kay et al., 2015). Cows calving at BCS >3.25 (on a 5-point scale; >5.5 on a 10-point scale) experience greater lipolysis and higher BHB and, therefore, risk of ketosis, increased hepatic lipid, and elevated acute-phase inflammatory markers (Akbar et al., 2015), whereas those calving at BCS ≤2.75 on a 5-point scale (or ≤4.5 on a 10-point scale) compromise milk yield, extend the postpartum anestrous interval, and increase disease susceptibility through impaired immune responses (Roche et al., 2009). Recent molecular data support these targets: grazing cows calving at medium BCS (e.g., 2.75 on 5-point scale, or 4.5 on 10-point scale) exhibit lower hepatic expression of inflammation-related genes and more favorable acute-phase and albumin–globulin profiles than cows calving at higher or lower BCS, consistent with optimized metabolic and immune function (Akbar et al., 2015).

Hypocalcemia remains an important mineral disorder in pasture-based dairy herds, but the grazing-specific literature supports a more cautious interpretation than is often drawn from housed-system studies (Roberts and McDougall, 2019). In 76 New Zealand spring-calving pasture-fed herds, the mean herd-level prevalence of subclinical hypocalcemia within 3 d of calving was 52%, with substantial between-herd variation, whereas mean farmer-reported clinical hypocalcemia prevalence was 2.9% (Roberts and McDougall, 2019). At the cow level, risk increased with age and was greatest on the day of calving, declining by 1 to 2 d postpartum, indicating that the period immediately around calving is the key window of hypocalcemia risk in grazing herds (Roberts and McDougall, 2019). Pasture-based feeding systems remain relevant to calcium homeostasis because precalving diets in Australian and New Zealand grazing systems can vary widely in DCAD, and in an indoor experiment using pasture hay plus freshly cut pasture, lowering precalving DCAD increased urinary calcium:creatinine ratio and increased magnesium absorption and renal excretion, responses consistent with improved calcium availability around calving (Roche et al., 2003). However, a New Zealand field study demonstrated that higher herd-level prevalence of subclinical hypocalcemia was associated with feeding grass silage or maize silage to precalving cows and with increasing amounts of supplemented magnesium oxide, which suggests that risk in grazing herds is influenced by the overall precalving feeding system rather than by any single supplement alone (Roberts and McDougall, 2019). Accordingly, this section is better framed around the documented prevalence and risk factors observed in pasture-fed herds, avoiding stronger claims about delayed hypocalcemia thresholds or blanket treatment strategies that are supported mainly by housed-system data (Roberts and McDougall, 2019).

One of the most consequential shifts in transition cow thinking over the past decade has been movement away from treating biochemical thresholds as diseases in themselves, particularly blood BHB and calcium concentrations, and, instead, evaluating them according to their relationships with outcomes such as milk yield, reproduction, and clinical disease. Historically, fixed blood BHB thresholds (e.g., ≥1.2 mmol/L for “hyperketonemia”) have been used to trigger treatment with gluconeogenic precursors such as monopropylene glycol, even in cows without clinical signs, based on the assumption that elevated BHB directly caused disease and that lowering BHB would improve health.

Recent work in pasture-based herds has challenged this assumption. In a large randomized controlled trial, Hendriks et al. (2025) identified that treatment of hyperketonemic cows with monopropylene glycol increased the probability of resolving hyperketonemia and lowered the risk of progression to severe hyperketonemia (BHB ≥3.0 mmol/L), but treated cows produced slightly less energy-corrected milk in the first 15 wk of lactation and showed no improvement in uterine health or reproductive performance. These results suggest that, in grazing systems, subclinical hyperketonemia without clinical signs is not a disease requiring routine therapeutic intervention.

Similar to hyperketonemia, the degree and duration of hypocalcemia is associated with hypocalcemia (total Ca ≤2.2 mmol/L) at 4 DIM is associated with reduced reproductive performance (Seely and McArt, 2023). Calcium supplementation (recommended 100 g/cow per day) in colostrum cows during the first 2 to 4 d postpartum through concentrate feeds, pasture dusting, or starter drenches provides effective hypocalcemia prevention (Roche et al., 2003). Despite subclinical hypocalcemia being identified as a significant problem, the value of routine blanket treatment with parenteral or oral calcium in grazing systems remains unclear to pasture-based dairy farmers (Horan et al., 2024). Accumulating evidence suggests that aggressive prevention through BCS, energy, and mineral management remains superior to reactive treatment and that calcium treatment is best reserved for cows with clinical signs or evidence of persistent low blood calcium beyond the first few days postpartum (McArt and Neves, 2020). This reorientation aligns with broader immunometabolism research, which emphasizes that metabolic challenges and their biochemical markers are part of an adaptive process (Horst et al., 2021; Zachut et al., 2022) and which supports focusing farm resources on correction of individual laboratory values.

Beyond macronutrient management, recent immunometabolism research has identified micronutrients and bioactive compounds that substantially modulate inflammatory and immune responses during transition. Omega-3 polyunsaturated fatty acids (n-3 PUFA), from both dietary sources and supplementation, serve as precursors for specialized proresolving mediators (SPM) that actively promote resolution of inflammation rather than merely suppressing it (Zachut et al., 2022). Fresh pasture naturally contains relatively high concentrations of n-3 PUFA, and marine lipid supplements can further enrich milk fat with eicosapentaenoic acid and docosahexaenoic acid in grazing dairy cows without consistently improving milk yield (Kitessa et al., 2004; Vahmani et al., 2014). Antioxidant micronutrients, including vitamin E, selenium, copper, zinc, and manganese, support immune cell function and reduce oxidative stress during the metabolic perturbations of transition (Chen et al., 2023). The specific requirements for these elements increase substantially during transition due to elevated metabolic demands. Recent research has also identified bioactive compounds in certain forages of grazing systems that may provide natural anti-inflammatory benefits for animal and human health (van Vliet et al., 2021). Organic forms of these elements demonstrate superior bioavailability and retention compared with inorganic sulfate or oxide forms, though economic considerations often dictate supplementation strategies in commercial operations (Fleming et al., 2025).

The rapid proliferation of precision livestock technologies, wearable sensors, accelerometers, computer vision systems, and artificial intelligence algorithms, has generated considerable enthusiasm about the potential to monitor individual cow transitions more closely and detect health problems before clinical signs become apparent. These technologies can track feeding behavior, rumination patterns, activity levels, body weight, and body condition, generating data streams that enable early-warning detection of metabolic or infectious disease (Eckelkamp and Bewley, 2020).

The potential value of these tools in grazing systems lies not in precision feeding of individual animals, a practical impossibility at pasture, but rather in detection of cows requiring closer monitoring or targeted intervention. For example, a wearable sensor might identify a cow with a sustained decrease in rumination time or feeding duration several days before clinical ketosis becomes manifest, enabling early supportive care and feed or mineral supplementation. Similarly, 3-dimensional computer vision systems that estimate BW and BCS could flag cows experiencing accelerated BCS loss postpartum, which has been demonstrated to be 3 to 4 times faster than visual body scoring (Albornoz et al., 2021). Further, the technology has prompted investigation for early disease or estrus detection (Eckelkamp and Bewley, 2020; de Oliveira et al., 2024). The practical utility of these technologies depends critically on their integration with farm management systems and veterinary decision-making; a perfect detection algorithm is of little value if managers lack capacity or clear protocols to act on alerts.

Current limitations significantly constrain adoption of wearable devices in grazing systems. They must withstand harsh pastoral environments, and battery life must extend to practical replacement intervals. More fundamentally, the sensitivity and specificity of sensor-generated alerts require validation across diverse grazing systems and environmental conditions, validation work that remains incomplete (Eckelkamp and Bewley, 2020). Three-dimensional camera systems, although promising for continuous BCS monitoring, have not yet been validated in grazing settings. Moreover, the cost per cow of precision monitoring infrastructure remains high, creating economic barriers to adoption in lower-margin grazing systems. Integration of genomic data with sensor data offers longer-term potential for identifying and breeding cows with superior metabolic resilience and immune competence (Thompson-Crispi et al., 2014; Džermeikaitė et al., 2025), yet longitudinal field data on the effects of genomic selection for these traits in grazing herds remain limited.

Despite these constraints, the trajectory of technology development and cost reduction suggests that targeted adoption of precision monitoring will become viable in grazing systems within the next 5 to 10 years, particularly for early-lactation disease detection. The key to success will be designing interventions that account for the realities of pasture systems, larger herd sizes, simpler facilities, and variable labor, rather than applying confinement-system protocols directly to grazing contexts.

Transition cow nutrition practices have underappreciated but substantial environmental and sustainability implications. Metabolic diseases during transition are associated with reduced feed efficiency, prolonged recovery to positive energy balance, and extended intervals to pregnancy, collectively resulting in more days of life required per unit of milk and dairy product. This extended timeline increases per-unit environmental impact (carbon footprint, nutrient excretion, land use). Conversely, improved transition management that reduces metabolic disease incidence, supports faster reproductive recovery, and extends productive longevity substantially improves environmental metrics on a per-unit-production basis (Ahsin et al., 2025).

Soil health and pasture management further influence transition cow nutrition through effects on the nutritive and mineral values of grazed forages. Soil mineral balance, or soil mineral stoichiometry, is influenced by soil C and the soil's cation exchange capacity, which, when understood, can help avoid animal health issues and transition outcomes (McAllister et al., 2020). For example, high pasture potassium can reduce dietary absorption of magnesium and induce a deficiency, increasing risk of grass staggers (McCarthy et al., 2025). This highlights the need to understand how soil and pasture diversity interact to support nutritious, resilient forage for the transition dairy cow. Integrating soil health, pasture diversity, and transition cow nutrition into a unified farm management strategy offers substantial long-term advantages for both production and sustainability. However, research quantifying these interactions and providing practical guidance for implementation remains limited and represents an important priority for future work.

Despite advances in understanding transition physiology and management, several critical knowledge gaps remain particularly relevant to pasture-based systems. First, the effectiveness of fatty acid supplementation may depend on pasture composition, but whether it provides a clear advantage requires further investigation across different pasture types and seasons in grazing systems. Second, practical immunomodulation strategies that account for environmental pathogen exposure and dietary variability in grazing systems need development and validation. Third, integration of precision sensor technologies for use in grazing systems, which experience variable pasture quality and intake patterns, to enable meaningful individual cow management in grazing systems, remains an unsolved technical and operational challenge. Fourth, quantification of the economic and environmental benefits of improved transition management, through reduced disease, enhanced productivity, and extended longevity, across diverse grazing farm types is needed to justify investment in more intensive transition management strategies. Fifth, scaling up research experiments to commercial herds needs to be undertaken to understand the pragmatism of recommendations and whether they result in improvements in cow health and production.

Emerging research opportunities include investigation of how pasture botanical diversity influences transition outcomes through provision of bioactive compounds and mineral concentrations; characterization of the interaction between soil mineral status and cow mineral nutrition across transition; and development of predictive models integrating individual cow genetics, behavioral phenotyping, and environmental factors to optimize transition outcomes. Work on genomic selection for metabolic resilience and immune competence in grazing populations is particularly important given the potential for long-term herd improvement.

The evidence synthesized here supports a hierarchical approach to transition management in grazing systems, with fundamentals prioritized ahead of refinements. Foundation practices include achieving and maintaining target BCS at calving (3.0 for mature cows, 3.25 for heifers; 5.0 and 5.5 on a 10-point scale, respectively) via strategic nutrition over the preceding 6 mo, implementing controlled prepartum energy intakes at 80% to 100% of ME requirements during the final 3 to 4 wk, and providing consistent magnesium and early postpartum calcium supplementation through practical delivery routes. Routine BCS monitoring at dry-off, mid-dry period, and 2 wk prepartum to ensure BCS targets are met, and 4 wk postpartum to capture postpartum BCS loss should guide feed budgeting and progress toward targets. Secondary measures, such as chelated trace minerals and targeted fatty acids, may add value when the fundamentals are already in place and budgets allow. Tertiary innovations include precision monitoring technologies, genomic indices incorporating metabolic resilience and immune competence, and advanced supplementation strategies for specifically identified problems.

Transition nutrition in pasture-based systems must remain pragmatic, emphasizing what is known, implementable, and economical, rather than adopting precision nutritional protocols as implemented in housed systems without adaptation. The core physiology and homeorhetic adaptations are conserved across systems, but the constraints of grazing require strategic modification rather than wholesale transfer of housed-cow programs. Evidence highlights 3 decisive control points: appropriate BCS at calving, controlled prepartum energy intake, and focused magnesium and calcium supplementation, which are widely achievable with moderate management discipline and should dominate extension and farm planning. Additional trace elements, specific fatty acids, and antioxidant strategies can be valuable add-ons, but sophisticated supplementation without solid condition and energy management remains analogous to building on sand.

Emerging data on subclinical metabolic markers encourage a shift away from treating laboratory thresholds as diseases and toward focusing on outcomes. Successful transition in grazing systems depends less on driving blood values toward reference ranges and more on managing a few critical levers that determine whether cows traverse the transition intact and enter a productive lactation. Precision technologies, genomics, and soil–plant–animal research offer genuine promise for improving transition management and long-term resilience, provided they are integrated thoughtfully into realistic grazing contexts. For farmers, advisors, and veterinarians, the way forward is to commit to BCS management, controlled prepartum energy linked to BCS, consistent mineral supplementation with practical methods, and monitoring for clinical disease while avoiding overinterpretation of laboratory cut-points. Within this fundamentals-first framework, evidence-based refinements can be added as economics and opportunity allow, offering the most dependable path to better transition outcomes, sustainable production, and long-term herd robustness in grazing systems.
